# Late-onset bloodstream infections of Very-Low-Birth-Weight infants: data from the Polish Neonatology Surveillance Network in 2009–2011

**DOI:** 10.1186/1471-2334-14-339

**Published:** 2014-06-18

**Authors:** Jadwiga Wójkowska-Mach, Ewa Gulczyńska, Marek Nowiczewski, Maria Borszewska-Kornacka, Joanna Domańska, T Allen Merritt, Ewa Helwich, Agnieszka Kordek, Dorota Pawlik, Janusz Gadzinowski, Jerzy Szczapa, Paweł Adamski, Małgorzata Sulik, Jerzy Klamka, Monika Brzychczy-Włoch, Piotr B Heczko

**Affiliations:** 1 Microbiology, Jagiellonian University Medical College, 18 Czysta Street, Krakow 31-121, Poland; 2 Clinic of Neonatology, Polish Mother's Memorial Hospital-Research Institute, Lodz, Poland; 3 Clinic of Neonatology and Intensive Neonatal Care, Warsaw Medical University, Warszawa, Poland; 4 Institute of Theoretical and Applied Informatics of Polish Academy of Sciences, Gliwice, Poland; 5 Division of Neonatology, Loma Linda University Children's Hospital, Loma Linda, California, USA; 6 Clinic of Neonatology and Intensive Neonatal Care, Institute of Mother and Child, Warszawa, Poland; 7 Department of Neonatal Diseases, Pomeranian Medical University, Szczecin, Poland; 8 Clinic of Neonatology, Jagiellonian University Medical College, Krakow, Poland; 9 Department of Neonatology, Poznan University of Medical School, Poznan, Poland; 10 Institute of Nature Conservation, Polish Academy of Sciences, Krakow, Poland

## Abstract

**Background:**

Late-Onset Bloodstream Infections (LO-BSI) continue to be one of the most important complications associated with hospitalization of infants born with very low birth weight (VLBW). The aims of this study were to assess the epidemiology of LO-BSI together with the risk factors and the distribution of causative pathogens at six Polish neonatal intensive care units that participated in the Polish Neonatology Surveillance Network from January 1, 2009 to December 31, 2011.

**Methods:**

The surveillance covered 1,695 infants whose birth weights were <1501 grams (VLBW) in whom LO-BSI was diagnosed >72 hours after delivery. Case LO-BSI patients were defined according to NeoKISS.

**Results:**

Four hundred twenty seven episodes of LO-BSI were diagnosed with a frequency of 25.3% and an incidence density of 6.7/1000 patient-days (pds). Results of our multivariate analysis demonstrated that surgical procedures and lower gestational age were significantly associated with the risk of LO-BSI. Intravascular catheters were used in infants with LO-BSI significantly more frequently and/or for longer duration: Central venous cathters (CVC) (OR 1.29) and Peripheral venous catheters (PVC) (OR 2.8), as well as, the total duration of total parenteral nutrition (13 vs. 29 days; OR 1.81). Occurrence of LO-BSI was significantly associated with increased the length of mechanical ventilation (MV) (OR 2.65) or the continuous positive airway pressure (CPAP) (OR 2.51), as well as, the duration of antibiotic use (OR 2.98). The occurrence of more than one infection was observed frequently (OR 9.2) with VLBW with LO-BSI. Microorganisms isolated in infants with LO-BSI were dominated by Gram-positive cocci, and predominantly by coagulase-negative staphylococci (62.5%).

**Conclusions:**

Independent risk factor for LO-BSI in VLBV infants are: low gestational age and requirement for surgery. The incidence rates of LO-BSI especially CVC-BSI were higher in the Polish NICUs surveillance than those of other national networks, similar to the central- and peripheral utilization ratio.

## Background

Late-Onset Bloodstream Infections (LO-BSI) continue as a critical complication associated with hospitalization of very low birth weight (VLBW) infants. LO-BSI contributes to morbidity, mortality, and other long-term adverse outcomes [1-5]. Surveillance of these infants, especially blood stream infections (BSI) was introduced in intensive care units in Poland [6,7] however, the epidemiology of these infections has not previously been collected systematically in Polish VLBW infants.

The epidemiology of infections among neonatal intensive care units (NICUs) in the USA has been explored through the National Healthcare Safety Network (NHSN) system of the Centers for Disease Control and Prevention (CDC); however, limited information has been available from the European countries. The biggest one, German “Krankenhaus-Infections-Surveillance-System”, NeoKISS (http://www.nrz-hygiene.de/en/surveillance/hospital-infection-surveillance-system) began in January 2000 as a prospective surveillance system for VLBW infants [8,9]. The similar surveillance systems have been implemented in France: Epidemiologie des Petits Ages Gestationnels, EPIPAGE study [10] and in the United Kingdom: the Neonatal Infection Surveillance Network, neonIN [11].

The aim of this study was to assess the epidemiologic factors and microbiological spectrum of primary LO-BSIs associated with or without intravascular devices together with identification of risk factors and the distribution of causative pathogens. A second aim was to implement uniform definitions, specimen acquisition, and culturing techniques for infection surveillance: continuous, systematic collection, analysis and interpretation of health-related and infections data.

## Methods

Utilization of data collected in the Polish Neonatology Surveillance Network (PNSN) for the scientific purpose was approved by the Bioethics Committee of Jagiellonian University Medical College – no. KBET/221/B/2011.

Continuous prospective target surveillance of infections was conducted from 1/1/2009 through 12/31/2011 at six Polish NICUs (only teaching hospitals) which participated in PNSN. These tertiary NICUs provided care for 20% of all VLBW infants born in Poland annually. The surveillance included infants hospitalized in these NICUs whose birth weights were <1501 grams (VLBW) at birth until they achieved a weight of 1800 grams or died. The general fatality case rate was 16.3%. The fatality case rate in subgroup of infants with a birth weight <500 grams was 75%; thus on 1 infant reached a weight of >1800 grams with a LO-BSI, and 3 were transferred to other units and were not further included in the surveillance within the PNSN. All VLBW infants of suspected or documented infected were subject to registration regardless of the time of occurrence according to criteria of Gastmeier et al. (see Appendix 1) when they had clinical signs of a bloodstream infection (BSI) [8]. Pneumonia and necrotizing enterocolitis were defined also according to criteria of Gastmeier et al. [8]. LO-BSI was defined when diagnosed >72 hours after delivery. Clinical sepsis represented an infant where signs of infection existed but on blood culture a causative organism was not identified. Central venous catheter (CVC)-associated BSI (CVC-BSI) and peripheral intravenous catheter (PVC) associated BSI (PVC-BSI) were infections associated with the use of a central or peripheral venous catheter within the preceding 48 hours prior to the onset of the infection [8,12]. The CVC utilization rate was 0.45 and the PVC utilization rates was 0.16 (calculated by dividing the number of days of CVC/PVC by the total number of patient days).

A surgical procedure was defined as surgery performed within an operating theater except for insertion of CVC involving a surgical incision for tissue removal or repair or to treat a surgical condition. The analysis was performed in infants prior their attaining a weight of 1800 grams in all cases. In the analysis of the relationship between LO-BSI and need for surgery, infants were included when the initial signs were observed between 3 to 30 days after operative intervention. Infants with more than one episode of infection were included; however, the addition of or change in pathogen was not necessarily indicative of a new episode of infection.

All blood specimens of at least 1 milliliter were injected into an aerobic blood culture bottle (Bactec Plus 26 Aerobic; BD Microbiology Systems) and cultured on MacConkey agar, horse blood agar (at 37 degrees C, each for 24 h) and Sabourand agar (at 37 degrees C for 38 hours. The isolates were characterized using biochemical tests, bioMerieux identification kit API system (bioMerieux, France).

### Statistical methods

These data were not subject to external validation (by researchers from other/external centers) and approximately 5% of the records were incomplete. Analysis of the impact of selected factors for the risk of LO-BSI was based on the group of infants who survived until the third day or longer. For the evaluation of the differences between the averages for the examined groups of infants, a one way analysis of variance (ANOVA) with the least significant difference test and Tukey’s test were applied. For the assessment of the frequency of infections in various groups of infants the chi-square test of independence was used.

A logistic regression model was used to initially examine whether there were observations with missing data or divergent data. The elimination of outliers for each variable was quantified based on box graphs, and we calculated measures of association using a model of selecting the explanatory variables intuitively understanding that each variable can have an effect on the dependent variable. The dependent variable was the dichotomous variable: the occurrence or non-occurrence of LO-BSI. Thereafter, a formal model of logistic regression was constituted to estimate the parameters using the quasi-Newton method. Analysis of the individual p-values allowed us to reject variables that were statistically insignificant. Before deciding to remove a variable, a logistic regression was performed separately for each factor (birth weight, gestational age, or requirement for surgery). The Clinical Risk Index for Babies (CRIB) score, and Apgar 1 and 5 minutes scores were also included in the logistic model. The odds ratio (OR) was used to determine the effect of a specific factor to increase the likelihood of LO-BSI.

A generalized linear model (GLZ) was applied to assess the significance of differences between *Staphylococcus aureus*, the coagulase-negative staphylocci (CNS), Enterobacteriaceae and other microorganism infection, and birth weight, gestational age, duration of hospitalization prior to the date of onset of the initial clinical sign or used of devices: CVC, PVC, mechanical ventilation (MV), and/or continuous positive airway pressure (CPAP). These models were evaluated separately for each bacterial groups. As the dependent variable was dichotomous, the binominal distribution was used. The predictors used in the model were continuous (birth weight, gestational age, duration of hospitalization), as well as, the categorical variables (use of CVC, PVC, MV and/or CPAP). Including such a set of variables in this model demands using the logit-linked function. Calculations were performed with the application of the open source library SciPy. A multivariate analysis of the influence of the risk factors on CVC-BSI identification was conducted using a classification tree learner [13,14] (Orange Biolab). A p <0.05 was considered statistically significant.

Our data were compared with NeoKISS surveillance system in Germany. In this analysis, data from one of six centers were not used because information about CVC and/or PVC catheterization, MV and/or CPAP was incomplete. NeoKISS data were chosen for comparison to the data from the PNSN owing to the meticulous research methods employed in this surveillance program.

## Results

### Description of the population

There were 1,695 newborns included in our surveillance of whom 1,243 who survived >72 hrs. This group was composed of 462 (27.3%) of extremely low birth weight infants with a birth weight < = 750gms, 313 (18.5%) infants with a birth weight from 751 to 999 gm, and 920 (54.3%) infants with birth weight ranging from 1000–1500 gm. Among these 1,234 infants, 164 (9.7%) underwent surgical procedures. The most common surgical procedure was closure of the patent ductus arteriosus (PDA) which represented 48.4% of all surgical procedures.

### Late-onset bloodstream infections

There were 427 episodes of LO-BSI diagnosed representing an incidence density of 6.7/1000 patient days (pds), including 46 cases of clinical sepsis were observed (10.7% of all LO-BSI). The onset of clinical signs of LO-BSI occurred an average of 15 days after birth (SD 14.3 days; median 11 days). Mean gestational age was 28 weeks (median 27 weeks) and the mean birth weight was 1100 grams (median 880 grams). Among infants with LO-BSI birth weights were significantly lower than in the group of newborns not having LO-BSI (Table 1).

**Table 1:** The characteristics of infants with and without symptoms of LO-BSI, who survived to the third day and/or later

	**Infant’s LO-BSI (-) [N = 816]**		**Infant’s LO-BSI (+) [N = 427]**		**Statistically significant P-value**
**Characteristics of patients**	**average; 95% CI**				
Gestational age [weeks]	29	(28; 29)	27	(27; 28)	<0.001
Gestational age infants who required surgery [weeks]	26	(26; 27)	25	(28; 29)	0.002
Gestational age infants who did not required surgery [week]	29	(29; 29)	28	(27; 28)	<0.001
Birth weight [grams]	1 114	(1 087; 1 141)	947	(920; 974)	<0.001
CVC [days]	15	(14; 16)	31	(27; 34)	<0.001
PVC [days]	9	(8; 10)	18	(16; 21)	<0.001
Mechanical ventilation [days]	12	(10; 13)	26	(22; 30)	<0.001
CPAP [days]	9	(8; 9)	16	(14; 17)	<0.001
Total parenteral nutrition [days]	13	(12; 14)	29	(27; 32)	<0.001
surgery in infancy*[No./%]	87	10.7	77	18.0	<0.001
Completion of the surveillance					
Death of the patient [No./%]	67	8.2	32	7.5	0.4
Transfer [No./%]	101	12.4	48	11.2	0.2
Body weight gain [No./%]	646	79.2	345	80.8	0.9

* up to 30 days after surgery; CI – confidence interval; LO-BSI, Late-Onset Bloodstream Infections; PROM, premature rupture of membranes; CVC, central venous catheter; PVC, peripheral intravenous catheter; CPAP, continuous nasal positive airway pressure.

The incidence of LO-BSI among infants within various birth weight categories were significantly different. The highest incidence rate (compared to infants with higher birth weight) was confirmed in the infants with birth weights <750 grams: 44.6% vs. 27.1% (OR 2.36; 95% CI 1.60-3.47). Results of the multivariate analysis found that low gestational age (p = 0.002; OR 3.9; 95% CI 2.99-5.12) and requirement for surgery (p<0.001; OR 1.8 95% CI 1.32-2.56) were significantly associated with risk of LO-BSI (up to 30 days after surgery) and others (Table 2).

**Table 2:** Crude and adjusted odds ratio (OR) and 95% confidence intervals (95% CI) for nevborns with and without symptoms of LO-BSI (late onset bloodstream infections), who survived to the third day and/or later (2009–2011)

	**Infant’s LO-BSI (-) [N = 816]**		**Infant’s LO-BSI (+) [N = 427]**	
**Characteristics of patients**	**crude OR**	**95% CI**	adjusted* OR	**95% CI**
Gestational age <28 week	3.9	2.99-5.13	71.808	71.57-72.05
Birth weight < 750 grams	2.362	1.60-3.48	3.732	3.45-4.01
Chorioamnionitis	1.404	0.80-2.45	1.185	0.96-1.42
PROM	1.079	0.78-1.48	3.343	2.97-3.72
CVC	1.293	0.93-1.79	2.886	2.49-3.27
PVC	2.803	2.06-3.81	1.523	1.14-1.91
Mechanical ventilation	2.645	1.97-3.56	12.642	12.31-12.98
CPAP	2.514	1.83-3.44	2.927	2.62-3.24
Total parenteral nutrition	1.813	1.25-2.64	2.912	2.72-3.11
Surgical procedures	1.882	1.28-2.77	2.912	2.72-3.11

Adjusted for birth weight; gestational age; surgery in infancy; Clinical Risk Index for Babies, CRIB; score, Apgar 1- and 5-minute scores.

PROM, premature rupture of membranes, CVC, central venous catheter; PVC, peripheral intravenous catheter; CPAP continuous nasal positive airway pressure.

Those infants with LO-BSI were more frequently vulnerable to other infection such as pneumonia (OR 2.13; 95% CI 1.52-2.97) or necrotizing enterocolitis (OR 1.55; 95% CI 1.00-2.44). The occurrence of more than one infection was observed frequently (OR 9.2; 95% CI 6.05-13.98). In the group of infants with signs of LO-BSI, we did not observe an increased mortality rate; however, early mortality associated with LO-BSI occurred usually < 7 days after the occurrence of the initial signs of LO-BSI was 7.5%.

The requirement for mechanical ventilation (OR 2.64 95% CI 1.96-3.55) or the use of CPAP (OR 2.51; 95% CI 1.83-3.44) were significantly more frequent among VLBW infants with LO-BSI. Analysis showed no association between the studied NICUs and the incidence rate of LO-BSI.

### Central and peripheral venous catheter-associated bloodstream infections

The CVC-BSI incidence rate was 8.6/1000 CVCdays, and the PVC-BSI incidence was: 10.5/1000 PVCdays. The incidence rate of PVC-BSI and CVC-BSI in infants with birthweights of 1000–1499 grams was significantly higher than in other groups of infants (RR 1.9, p<0.001). In infants with LO-BSI, compared to infants without LO-BSI, the catheters were used significantly more frequently and/or for a longer time: CVC (OR 1.2931, 95% CI 0.93-1.79) and PVC (OR 2.8031, 95% CI 2.06-3.81) (Table 1). Short-term use of PVC – up to 10 days – did not influence on the risk of LO-BSI.

Also, in infants with LO-BSI compared to infants without LO-BSI, total parenteral nutrition was prescribed significantly more frequently (OR 1.813, 95% CI 1.25-2.64), and/or for a longer duration, average: 13 vs. 29 days (over 16 days, OR 4.3489, 95% CI 3.17-5.96).

### Microbial aetiology of the LO-BSI

Gram-positive cocci constituted 77% of the isolated pathogens from CVC-BSI. The largest group was coagulase-negative staphylococci (CNS) and *Staphylococcus aureus*. The dominant species among group of the CNS isolates were *S. epidermidis* (62.5%), then *S. haemolityicus* (20%), *S. warneri* (7.5%), *S. hominis* (5%), *S. xylosus* (2.5%) and *S. capitis* (2.5%).

Gram-negative rods were isolated more often in PVC-BSI than in CVC-BSI (Table 3), especially, *Klebsiella spp* which were the second most common microorganisms in PVC-BSI. Infections caused by yeast-like fungi constituted 3.8% of all the BSI cases and there were no other fungal infections.

**Table 3:** The occurence of the pathogens of the late-onset BSI, diagnosed >72 hours after delivery (2009-2011)

	**CVC-BSI [N = 208]**		**PVC-BSI [N = 74]**		**All LO-BSI**		**Fatal cases No./ fatal care rate**
	**No/%**						
**Gram-positive microorganisms**	**180**	**81.8**	**54**	**72.0**	**304**	**77.0**	
CNS	143	65.0	41	54.7	247	62.5	6 / 2.4%
*Staphylococcus aureus*	18	8.2	7	9.3	31	7.8	1 / 3.2%
*Enterococcus spp./Streptococcus spp*	19	8.6	6	8.0	26	6.6	1 / 3.8%
**Gram-negative microorganisms**	**32**	**14.5**	**17**	**22.7**	**73**	**18.5**	
*Escherichia coli*	12	5.5	5	6.7	26	6.6	4 / 15.4%
*Klebsiella spp.*	11	5.0	9	12.0	27	6.8	3 / 11.1%
*Enterobacter spp*.	3	1.4	2	2.7	8	2.0	
*Enterobacteriaceae* other	1	0.5	1	1.3	4	1.0	2 / 50%
*Acinetobacter baumanii*	0	0.0	1	1.3	3	0.8	
*Pseudomonas aeruginosa*	5	2.3	0	0.0	5	1.3	4 / 80%
Yeats	8	3,6	3	4.0	15	3.8	1 / 6.7%
Others	0	0.0	1	1.3	3	0.8	
Total*	**220**	**100.0**	**76**	**100.0**	**395**	**100.0**	

Legend:* Total microbial etiological factors is greater than the number of LO-BSI confirmed microbiologically by a mixed culture found in several infections.

LO-BSI – late onset bloodstream infections, CVC-BSI – Central Venous Catheter-Associated BSI, PVC-BSI Peripheral Venous Catheter-Associated BSI; CNS – *Coagulase-negative staphylococci.*

There was no relationship between the bacterial etiology of LO-BSI, and the following: birthweight, gestational age, length of hospitalisation before the date of first signs and the use of devices analysed separately with one-factor statistical techniques.

## Discussion

Our results of LO-BSI are the first to be reported by the Polish Neonatology Surveillance Network (PNSN) and from Central Europe (except Neo-KISS) based on a national program for infection surveillance and control among NICUs. Our previous report on this population within the PNSN focused on early onset infection [15].

LO-BSI incidence in our population are lower than those observed in other national surveillance projects. For example, in a report from the Netherlands the incidence in their groups of 742 VLBW infants was 14.9/1000 pds [16], and in the German NeoKISS study in their first year of reporting their incidence was 8.3/1000 pdys that included 303 VLBW infants. In the USA, the reported incidence for infants born at 28 weeks gestation or earlier was: 36% [1], while in Israel a 39% rate [17].

Our data focusing on infections associated with vascular lines do differ substantially from those published by other centers e.g. NeoKISS [18]. The CVC-BSI in our total study population was greater than 8.6/1000 CVC days, while in the NeoKISS surveillance it was 13.8/1000 CVC days during the initial year of reporting. However, in our population we did not identify CVC-BSI in extremely low birthweight infants (ELBW) infants <499 grams, because of their high fatality rate. However, in the NeoKISS surveillance program this group had the highest risk of CVC associated infections, as well as, PVC-BSI. Unfortunately, this severe morbidity in Polish NICUs had the highest incidence of both CVC-BSI and PVC-BSI among infants with birth weights <1000 gms. Our surveillance found a 60-80% increase over than reported by the NeoKISS program [9], and higher than that reported by Sengupta et al. [18].

Therefore, our results indicate the need to reduce the risk of infections associated with vascular line in Polish NICUs; Gastmeier et. al. found that after 3 years of surveillance: German NICUs reported a significant reduction in the of CVC-BSI infants from 13.8 to 10.6 cases/1000 CVC days (RR. 0.77, p=0.008) [9,19]. The German experience suggests that participation in ongoing surveillance of nosocomial infections in NICUs, requiring individual units to provide data may lead to a reduction in BSI rates when there is through participation in a national surveillance system with feedback and quality improvement [19]. Historical data from the American National Healthcare Surveillance Network [20] from 1992–2001 found that CVC- BSI was 11.3/1000 CVCdays among infants with birth weights <750 gm and 6.9/1000 CVCdays among infants 1001–1500 gm. However, the most current data from this surveillance network reported a 4 fold reduction in catheter associated infections in 2012 [21], and similar to rates reported from Canada [22].

The next important issue: our data also indicate a high CVC utilization rate in Polish NICUs: almost twice as likely and often (or: for a longer duration) than in the German NICUs [9].

Gram positive cocci organisms were the dominant microorganisms isolated in LO-BSI, including CNS, and especially associated with catheter associated infections. The predominance of these organisms were associated with catheter related LO-BSI in the USA [18], Japan [23], Taiwan [24] and Israel [25]. Freeman et al. first described dominance of CNS with increasing survival of ELBW infants in the 1970s [26] with longer duration of use of intravenous catheters [26]; fortunately, LO –BSI with CNS have not been characterized by an increased mortality [26,27].

Gram-negative rod infections were mostly observed among LO-BSI in patients without prolonged use of vascular lines; however, we found a lower frequency of gram-negative rod infections than have been reported in the UK [11], Taiwan [24], Germany [9], or U.S.A. [28]. In our surveillance yeast LO-BSI infections were found in 3.8% of infants <1000 gm and lower than that reported by Greenberg [3,29], but higher than that reported by Mazoni (1.4%) [30].

VLBW infants constitute a small group of patients undergoing surgical procedures. The literature on postsurgical in VLBW newborns has not been widely publicized. Many researchers studying the epidemiology of nosocomial infections exclude the group of VLBW infants undergoing surgery citing the diversity of this specific population that would interfere with more generalizable knowledge. In our studies the dominant problem were LO-BSI associated with infant surgery. In a publication from the U.S., risk factors for infections are late PDA closure procedures (6 or more days after diagnosis) and very low birthweight [31]. Importantly, the number of PDA closures (81 in 1243 infants who survived > 72 hrs) is consistent with the report by Evans, who found about 10% of infants born before 27 weeks (and 3% of infants born at 27–29 weeks) required PDA surgical ligation [32]. Small number reports about the state of post-operative infants is insufficient [33,34].

A limitation of this study is that there were not independent auditors at each site to valid submitted data. Furthermore, VLBW infants frequently have multisystem diseases resulting from immaturity such as respiratory distress syndrome and apnea or bradycardia episodes that require interventions with medical devices (MV or CPAP). For the first weeks of life limited enteral nutrition must be supplement with parenteral nutrition provided through vascular catheters that also increase their risk for infection. Infant born after maternal chorioamnionitis may receive initial antibiotic therapy that is of insufficient duration to treat neonatal sepsis, or may not have sufficient spectrum of coverage to treat organisms more prevalent in LO-BSI. As a group VLBW infants are immunologically "immature" with limited and variable transplacental antibody transfer from their mothers further increasing their risk for LO-BSI. Nonetheless by introducing common definitions, technique and quantity of blood specimen acquisition, and uniform microbiological techniques at each center in the PNSN, our surveillance provide for baseline data for quality improvement initiatives. Our focus will be to reduce the duration of CVC and PVC use to minimize the risk of LO-BSI in this population, and to meticulously care for these catheters once inserted.

## Conclusions

In VLBW infants in whom use of CPAP or mechanical ventilation is nearly universal and in whom the presence of vascular catheters is nearly routine, we found that coagulase-negative staphylocci were the most common cause of LO-BSI. While rates of LO-BSI especially associated with CVC- BSI were higher in the Polish NICUs than those of other national networks, we now have evidence to inform the neonatologist into more precise selection of antimicrobial coverage when an LO-BSI is suspected. Furthermore, based on these findings it is necessary to implement a national program of infectious disease monitoring while promoting the shortest duration of CVC and PVC use as possible. There is also an ongoing need to monitor all VLBW undergoing surgery in the neonatal period for post-surgical infections to reduce the LO-BSI incidence.

## Appendix 1

Definitions of infections in infants born with very low birth weight, according to [8]:

1. Bloodstream infection (BSI):

• Presence of at least two of the following: temperature >38°C or <36.5°C or temperature instability, tachycardia or bradycardia, apnea, prolonged capillary refill, metabolic acidosis, hypoglycemia, other signs of bloodstream infections such as lethargy;

and

• Physician already having instituted treatment for sepsis for at least five days; blood culture not done or no organism detected in blood; no apparent infection at another site (clinical sepsis)

or

• Recognized pathogen cultured from one or more blood cultures or cerebrospinal fluid and no coagulase-negative staphylococci (pathogen not related to an infection at another site) or coagulase-negative staphylococci isolated from at least one blood culture or intravascular line and one of the following: C-reactive protein >2.0 mg/dL, immature/total neutrophil ratio (I/T ratio) >0.2, leukocytes <5000/μl, platelets <10000/μl.

2. Pneumonia:

• one of the following criteria: chest radiography showing new or progressive infiltrates, consolidation or fluid in the lobar fissures/pleura, and

• worsening of gas exchange, and

• four or more of the following signs: worsening of gas exchange, temperature instability, new onset or increasing bradycardia (<80/min) or new onset or increasing tachycardia (>200/min), new onset or increasing tachypnea (.60/min) or apnea, (>20 s), new onset or increasing dyspnea (retractions, nasal flaring, grunting, increasing production of respiratory secretions and increasing need for suction, purulent tracheal secretions, isolation of a pathogen in respiratory secretions, C-reactive protein2.0 mg/dl, immature/total ratio of neutrophil blood cells 0.2 or more)

3. Necrotising enterocolitis (NEC):

• Presence of two of the following clinical signs and symptoms without any other recognized reason at least two of the following signs: vomiting, abdominal distention, pre-feeding residuals, redness of flanks, persistent microscopic or gross blood in stools;

• and at least one of the following criteria: pneumoperitoneum, pneumatosis intestinalis, unchanging ‘rigid’ loops of small bowel; or histological evidence of NEC.

## Competing interest

The authors declare that they have no conflict of interest.

## Authors’ contributions

JWM, EH and PBH designed the Polish Neonatology Surveillance Network (PNSN), JWM analyzed and interpreted the data and wrote the manuscript; TAM supported the study and wrote the manuscript; EG, MN, MBK, EH, AK and DP, EG, JG, JS, JK and MS conceived of the study, participated in its design and coordination, collected the data about VLBW neonates; JD and PA performed the statistical analysis. MBW financially supported the study. All authors read and approved the final manuscript.

## Pre-publication history

The pre-publication history for this paper can be accessed here:

http://www.biomedcentral.com/1471-2334/14/339/prepub
